# Prognostic utility of chromosomal instability detected by fluorescence *in situ *hybridization in fine-needle aspirates from oral squamous cell carcinomas

**DOI:** 10.1186/1471-2407-10-182

**Published:** 2010-05-06

**Authors:** Hiroaki Sato, Narikazu Uzawa, Ken-Ichiro Takahashi, Kunihiro Myo, Yoshio Ohyama, Teruo Amagasa

**Affiliations:** 1 Maxillofacial Surgery, Maxillofacial Reconstruction and Function, Division of Maxillofacial and Neck Reconstruction, Graduate School, Tokyo Medical and Dental University, Tokyo, Japan

## Abstract

**Background:**

Although chromosomal instability (CIN) has been detected in many kinds of human malignancies by means of various methods, there is no practical assessment for small clinical specimens. In this study, we evaluated CIN in fine-needle aspiration (FNA) biopsied oral squamous cell carcinomas (SCCs) using fluorescence *in situ *hybridization (FISH) analysis, and investigated its prognostic significance.

**Methods:**

To evaluate CIN status of tumors, FISH with genomic probes for the centromeres of chromosomes 7, 9, and 11 was performed on specimens obtained by FNA from 77 patients with primary oral SCCs.

**Results:**

High-grade CIN (CIN3) was observed in 11.7% (9/77) of patients with oral SCCs and was associated significantly with reduced disease-free survival (*p *= .008) and overall survival (*p *= .003). Multivariate Cox proportional hazards analysis showed that CIN status was significantly correlated with disease-free survival (*p *= .035) and overall survival (*p *= .041).

**Conclusion:**

Analysis of CIN status using FISH on FNA biopsy specimens may be useful in predicting of recurrence and poor prognosis in patients with oral SCCs.

## Background

Head and neck squamous cell carcinoma (HNSCC) is a common malignancy, accounting for an estimated 274,289 new cases and 127,459 deaths worldwide in 2002, or roughly 2.5% of all new cases of cancer and 1.9% of all cancer deaths annually [1]. Over 50% of HNSCCs arise in the oral cavity. Although advances in surgical techniques, radiotherapy and chemotherapy have improved the extent of organ preservation and overall quality of life, as well as reducing morbidity, disease-free survival rates (DFS) and overall survival rates (OS) for patients with HNSCC have remained largely unchanged over the past 20 years [2]. To improve the long-term survival rate of patients with HNSCC, it is important to find more accurate prognostic markers to aid the selection of more appropriate treatment.

It is now widely accepted that nearly all solid tumors are genetically unstable. Genetic instability has been shown to comprise two forms: microsatellite instability (MIN) and chromosomal instability (CIN). MIN, which is known to be a genetic phenotype of non-polyposis colorectal cancer, is observed at the nucleotide level [3]. In HNSCC, the reported frequencies of MIN vary from 1.23 [4,5] to 57.9% [6]; in general the incidence is relatively low. In contrast, in most solid tumors including oral SCCs, CIN occurs at the chromosomal level, with frequent gains and losses of whole chromosomes or chromosomal segments. CIN has been assessed by various methods, including flow cytometry, karyotyping, comparative genomic hybridization (CGH), allelotyping, Inter-(simple sequence repeat) PCR, and genomic fingerprinting [7-12]. Although these methods are very informative, they are cumbersome for determining CIN, and therefore not practical for the assessment of clinical specimens. On the other hand, fluorescence *in situ *hybridization (FISH) has made it possible to detect numerical changes in chromosomes and genes easily and rapidly in small surgical samples, such as those obtained by fine-needle aspiration (FNA) biopsy. Therefore, FISH analysis is one of the most practical methods for evaluation of CIN in surgical specimens.

With regard to HNSCC and its subset, oral SCC, it is well known that the occurrence of the MIN phenotype is relatively low, and this is supported by the fact that many loss of heterozygosity (LOH) events seem to have been more common than MIN in HNSCC [4]. On the other hand, many previous studies have demonstrated that karyotypes of HNSCC and oral SCC consist of near-triploid chromosome numbers and contain various patterns of cytogenetic abnormality, including structural and numerical aberrations [12-14]. Moreover, these features have been confirmed by other molecular genetic techniques such as CGH, LOH, and FISH. These findings indicate that CIN rather than MIN is the dominant genetic event in carcinogenesis of oral SCCs and may play an important role in oral cancer progression.

In the present study, we examined CIN grade using FISH in FNA biopsy samples from primary oral SCCs, and analyzed the association between CIN status and clinical and histopathological factors. To our knowledge, the present study represents the first analysis of CIN grade in FNA biopsied oral SCC detected by FISH, and also the first to examine whether CIN status has any impact on clinical outcome.

## Methods

### Patients

Tissue samples were obtained from 77 patients (52 males, 25 females) with oral SCC who had undergone primary surgical excision with curative intent at the Maxillofacial Surgery, Graduate School, Tokyo Medical and Dental University (Tokyo, Japan), between April 2000 and October 2006. No patients received preoperative or postoperative treatment. Informed consent was obtained from all the patients in accordance with our Institutional Review Board guidelines. The mean age of the patients was 59.9 years (range, 20-89 years). The oral SCC samples were derived from the tongue (n = 42), lower gingiva (n = 22), upper gingiva (n = 4), buccal mucosa (n = 3), and the floor of the mouth (n = 6). The clinical staging was defined on the basis of the International Union Against Cancer TNM classification [15]: 20 patients were stage I [T1N0M0], 30 were stage II [T2N0M0], 12 were stage III [T3N0M0, T1-3N1M0], and 15 were stage IV [T4N0M0, anyTN2,3M0, anyTanyNM1]. The median follow-up period was 45.4 months (range, 6.1-105 months). Tumors were classified histopathologically as well, moderately, or poorly differentiated according to their cellular differentiation as defined by the World Health Organization criteria [16]. Disease-free survival (DFS) was calculated from the time of initial examination to the time of local, regional, or distant recurrence of the disease, or at the five-year follow-up. Overall survival (OS) was calculated from the time of initial examination to the time of death, or to the time of the five-year follow-up.

### FNA biopsy samples and FISH

Sampling of the tumor cells and slide preparation for FISH analysis were performed as described previously [17,18]. A suspension of single cells was obtained by aspirating the tumor with a 21-gauge needle. The cells were soaked in 0.05 M KCl solution for 2 min to disrupt the cell membranes and expose the naked nuclei, and then fixed by addition of an equal volume of methanol/acetic acid (3:1) solution (Carnoy). After centrifugation at 3000 rpm for 10 min, the upper layer was exchanged for Carnoy solution. Centrifugation and solution exchange were repeated twice, and the resulting upper layer was transferred dropwise to glass slides under steam. The specimens were then air-dried and stored at -20°C until use.

To detect changes in the copy number of chromosomes 7, 9 and 11 in the oral SCC cells, we used three types of single-color BAC clone probes specific for the centromeric DNA of these chromosomes. A single-color probe specific for chromosome 7 centromeric DNA was labeled with Spectrum Green (D7Z1 for CEP7, Vysis Inc., Downers Grove; IL), one for chromosome 9 centromeric DNA was labeled with Spectrum Orange (CEP9, Vysis Inc.), and one for chromosome 11 centromeric DNA was labeled with Spectrum Green (D11Z1 for CEP11, Vysis Inc.). Single-color FISH was carried out as follows. Briefly, the materials on the slides were aged in 2 × saline-sodium citrate (SSC)/0.1% (v/v) NP-40 at 37°C for 30 min and dehydrated through an ethanol series. The slides were denatured in 70% (v/v) formamide/2 × SSC at 75°C for 5 min and dehydrated through an ethanol series. The probe, denatured at 75°C for 5 min, was placed on the denatured slides, covered with Parafilm (American National Can, Greenwich, CT), and incubated in a humid box at 37°C overnight. After being washed at 45°C three times in freshly prepared 50% (v/v) formamide-2 × SSC for 10 min, SSC for 10 min, and 2 × SSC-0.1% (v/v) NP-40 for 5 min, the slides were counterstained with 4,6-diamidino-2-phenylindole, dihydrochloride (DAPI; 1 μg/ml).

### Fluorescence microscopy

An Olympus BX50 epifluorescence microscope equipped with × 60 and × 100 oil-immersion objectives and a triple-pass filter for Spectrum Green/Spectrum Orange and DAPI (Vysis Inc.) was used to count the fluorescent signals. Small, round lymphocyte-like cells, and overlapping and damaged nuclei were ignored, and only intact nuclei, especially large nuclei, were evaluated. Hybridization signals were counted in 100 interphase nuclei.

### CIN evaluation of FISH analysis

The modal copy number of each chromosome was determined [3]. The fraction of cells with chromosome numbers that differed from the mode (variant fraction: F) was calculated for each chromosome. The average variant fraction (F-AVG) for all three chromosomes (7, 9 and 11) was calculated as follow: F-AVG = (F7 + F9 + F11)/3, where F7, F9 and F11 are the variant fractions of chromosome7, 9 and 11, respectively. The degree of CIN was graded as follows: CIN1, the percent of cells with average nonmodal copy numbers (F-AVG) in < 20% of the cells; CIN2, F-AVG in ≧ 20%, < 40% of the cells; CIN3, F-AVG in ≧ 40% of the cells [19].

### Statistical analysis of FISH results

The results of single-color FISH were compared with clinicopathological information using the chi-squared test and two-tailed Fisher exact test. The clinicopathological information included patient age, gender, tumor site, clinical T stage, clinical N stage, histopathological grading, and disease stage. DFS and OS were calculated using the Kaplan-Meier method, and statistical significance was determined by log rank test. Multivariate disease-free and overall survival analyses were performed using the Cox proportional hazards model. The level of significance was set at *p *< 0.05. All statistical analyses were performed using SPSS 15.0J software (SPSS Inc., Chicago, IL).

## Results

### Modal copy number

**Figure 1:**
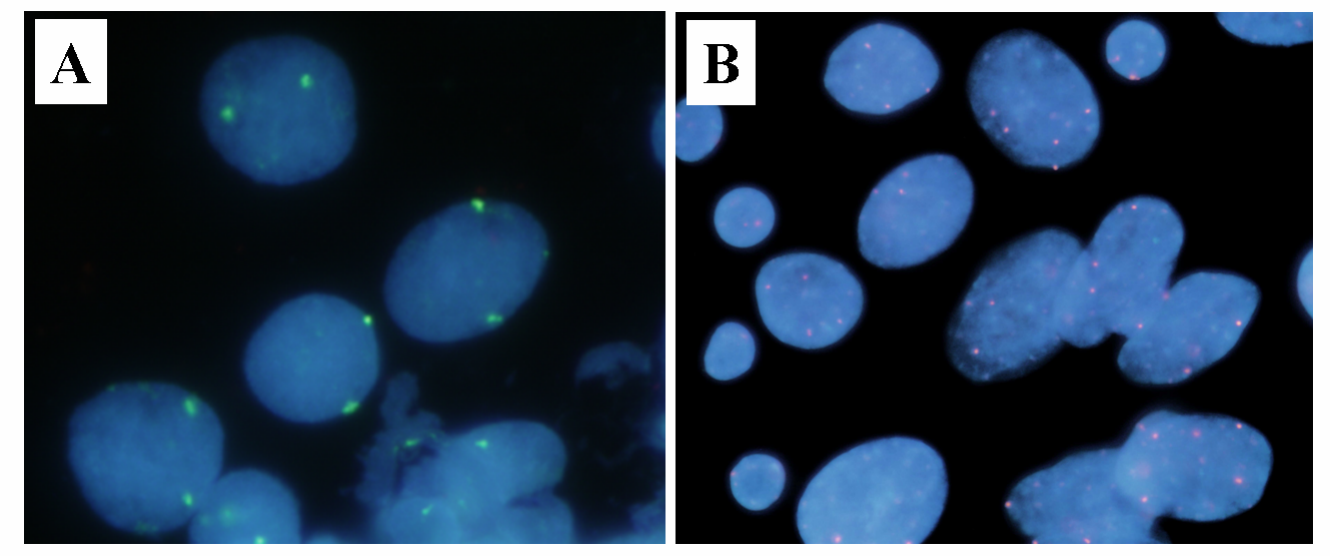
**Representative FISH analysis**. Cells were hybridized with probes for the chromosome 7, 9, or 11 centromere (green or orange). (A) A representative example of a normal lymphocyte; hybridized with single probe for the chromosome 11 centromere (green). FISH analysis revealed two copies of centromeric signal in all nucleus. (B) High-grade CIN case. Note the presence of heterogeneous copy number of the centromeric signal of chromosome 9.

The copy numbers of chromosomes 7, 9, and 11 were analyzed in 77 primary oral SCCs. The modal copy number of each chromosome ranged from two (disomy) to six (hexasomy), and the percentage of cells that did not carry the modal copy number was quite variable (Figure 1, Table 1).

**Table 1:** The prevalence of aneusomy in surgical specimens of oral SCC

	Chromosome no.			
		
Modal copy no.	7	9	11	Total (%)
Disomy	71	72	69	212 (91.8)
Trisomy	1	3	1	5 (2.2)
Tetrasomy	5	1	7	13 (5.6)
Pentasomy	0	0	0	0 (0.0)
Hexasomy	0	1	0	1 (0.4)

### Correlation between CIN grade in oral SCCs and clinicopathological parameters

CIN grade in the patients with oral SCC was determined by FISH with centromere repeat probes for chromosomes 7, 9, and 11. CIN1 was observed in 50 out of 77 cases (64.9%), CIN2 in 18 out of 77 cases (23.4%), and CIN3 in 9 out of 77 cases (11.7%).

The correlations between CIN grade and clinicopathological parameters in the patients with oral SCC are summarized in Table 2. CIN grade was not correlated significantly with patient age, gender, tumor site, disease stage, clinical T stage, clinical N stage, or recurrence. However, poorly differeciated tumor was associated significantly with high-grade CIN (CIN3, chi-squared test, *p *= .048). Moreover, CIN3 was significantly correlated with cancer death (*p *= .013).

**Table 2:** Correlation between CIN status and Clinicopathological Parameters

Clinicopathological Parameters	CIN			*p *value^a^
		
	Grade 1	Grade 2	Grade 3	
Age (yrs)				
<60	21	6	5	
≧60	29	12	4	NS
Gender				
Male	32	12	8	
Female	18	6	1	NS
Tumor site				
Tongue	26	11	5	
Lower gingiva	15	4	3	
Upper gingiva	4	0	0	
Buccal mucosa	2	0	1	
Floor of mouth	3	3	0	NS
Disease stage				
I, II	34	12	4	
III, IV	16	6	5	NS
Cellular differentiation^b^				
Well to Moderate	47	15	6	
Poor	3	3	3	*p *= .048
Clinical T stage				
1, 2	40	15	5	
3, 4	10	3	4	NS
Clinical N stage				
0	41	12	5	
1 to 3	9	6	4	NS
Recurrence	10	6	5	NS
Cancer death	7	6	5	*p *= .013

NS: not significant

^a ^By chi-square test and two-tailed Fisher exact test

^b ^Histopathologic diagnosis

### Association between CIN grade in oral SCCs and survival

**Figure 2:**
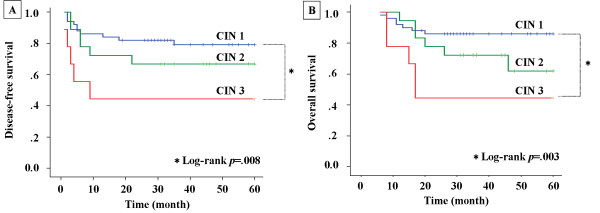
**Disease-free and Overall survival of 77 patients of oral SCCs according to CIN status**. **A**, Kaplan-Meier curve for disease-free survival according to CIN status. **B**, Kaplan-Meier curve for overall survival according to CIN status.

Kaplan-Meier survival curves for DFS and OS are presented in Figures 2A and 2B, respectively, and clearly demonstrate the adverse impact of high-grade CIN (CIN3) compared with low-grade CIN (CIN1) on both disease recurrence (log rank test, *p *= .008) and OS (log rank test, *p *= .003). Multivariate analysis including clinicopathological factors (age, gender, cellular differentiation, and disease stage) and CIN status (CIN3 versus CIN1 and CIN2) revealed that two factors, disease stage and CIN status, predicted a poor outcome in DFS and OS (Table 3).

**Table 3:** Multivariate Cox Proportional Hazards Analysis on CIN status

	Disease-free survival			Overall survival		
		
Clinicopathological Parameters	*p *value	Risk ratio	95%CI	*p *value	Risk ratio	95%CI
Age	NS	-	-	NS	-	-
Gender	NS	-	-	NS	-	-
Cellular differentiation	NS	-	-	NS	-	-
Disease stage	0.014	3.236	1.272-8.234	0.002	5.845	1.950-17.517
CIN	0.035	3.478	1.091-11.088	0.041	3.708	1.057-13.003

CI: confidence interval

NS: not significant

## Discussion

It has been recognized that tumors arise and progress as a result of multiple genetic alterations, such as chromosomal aberrations, DNA changes (e.g., mutations, amplifications, or deletions), and/or mRNA alterations through epigenetic changes [14]. In HNSCCs including oral SCCs, exposure to environmental agents, such as tobacco smoke, alcoholic beverages, and viruses, including human papillomavirus, may affect the process of carcinogenesis [2,20]. Previous studies have demonstrated that the karyotypes of human oral SCC cells are near-triploid and contain numerical and structural chromosomal abnormalities including balanced and unbalanced translocation, deletions, dicentric chromosomes, gene amplification in the form of extra-chromosomal double minutes or intra-chromosomal regions of homogeneous staining [14]. Although these chromosomal aberrations are clonal genetic changes, many numerical and structural variations were detected in the oral SCCs, suggesting that this kind of cancer exhibits a high rate of CIN, and that CIN may play an important role in their tumorigenesis. In this study, we investigated CIN status using FISH on FNA biopsy samples from primary oral SCCs, and analyzed the association between CIN grade and clinical and histopathological factors. Our findings clearly demonstrated that CIN grade may be a significant predictor of recurrence and poor outcome in patients with this malignancy.

CIN has been observed in almost all solid tumors and studied by various methods, including FISH. The relationship between CIN status determined by FISH and poor prognosis has been studied in a number of human malignancies, including lung cancer [21], malignant astrocytic tumor [22], and lymphoma [23]. These studies have demonstrated that CIN status can be effectively detected using FISH analysis and is significantly associated with poor prognosis. For example, Nakamura et al. reported that 28% of non-small cell lung cancers they investigated had heterogeneity of all four chromosomes (chromosomes 3, 10, 11, and 17) examined, and were judged to be carrying CIN [21]. Using univariate and multivariate analysis, they also showed that CIN was strongly associated with a worse prognosis, suggesting that CIN can be considered an independent indicator of poor prognosis in non-small cell lung cancer. With regard to head and neck cancers, including oral SCC, a number of studies have investigated numerical chromosomal alterations by means of FISH. Hardisson et al. characterized numerical aberrations in routine surgical specimens of 50 primary human SCCs of the pharynx and larynx using FISH with DNA probes specific for the centromeric sequences in chromosomes 8, 9, 11, and 17 [24]. They showed that numerical abnormalities of these chromosomes were present in the vast majority (92%) of these cancers. Although they clearly demonstrated an overall positive correlation between DNA ploidy determined by DNA flow cytometry (FCM) and numerical chromosomal aberration determined by FISH, they did not examine the relationship between chromosomal status and clinical and histopathological factors including recurrence and survival. Soder et al. analyzed numerical chromosomal changes during the progression of HNSCC from low-stage non-metastasizing tumors to high-stage metastasizing tumors and lymph node metastasis using the FISH technique with 6 centromeric DNA probes (for chromosomes 1, 7, 9, 11, 17, and 18) [25]. They demonstrated a high correlation (*p *<.0001) between tumor metastatic potential and aneuploidy. Unfortunately, however, they did not examine whether these chromosomal aberrations have any impact on the clinical outcome of this cancer. Moreover, Bergshoeff et al. have reported that CIN detection by FISH employing two centromeric DNA probes (foe chromosomes 1 and 7) in oral SCC is strongly associated with regional tumor outgrowth (p = .018) [26]. However, they also did not demonstrate a correlation between numerical chromosomal changes and prognosis. Thus, although these studies investigated numerical chromosomal aberrations in oral SCC using FISH with DNA probes specific for several centromeric sequences, they did not examine the correlation between CIN, clinical and histopathological factors, and outcome. In the present study, therefore, to establish a simple and practical method for evaluation of CIN grade in primary oral SCCs, we selected only three chromosomes 7, 9, and 11 and investigated their numerical aberrations using FISH for interphase nuclei obtained by FNA biopsy, together with the possible impact of CIN status on clinical outcome.

We demonstrated that high-grade CIN was significantly correlated with poorer outcome (OS) by univariate (*p *= .003) and multivariate analysis (*p *= .041). Why, then, is CIN status in oral SCC associated with a poorer prognosis? There are at least two possible answers. First, in general, CIN may induce accumulation of genetic alterations. CIN gives rise to an increased rate of loss or gain of whole chromosomes or large parts of chromosomes during cell division. Consequently, this may result in an imbalance of chromosome number (aneuploidy) and an increased rate of loss of LOH [27]. These genetic alterations may easily accelerate changes in the expression of many types of cancer-related genes such as oncogenes and tumor suppressor genes. Therefore, genetic alterations of genes that may contribute to acquisition of a malignant phenotype are more frequent in cancer cells with high-grade CIN than in those without. Another possibility is that abnormalities of several significant genes located on chromosomes 7, 9, and 11 may affect the progression of oral SCCs. Epidermal growth factor receptor (*EGFR*), p16 (*CDKN2A*), and cyclin D1 (*CCND1*), which may play an important role in the tumorigenesis and progression of this cancer, are located on chromosomes 7, 9, and 11, respectively. Our previous studies clearly demonstrated that numerical aberrations of these genes are a reliable predictor of recurrence and outcome in oral SCCs [17,18,28-30]. We have tried to compare chromosome number aberrations (this study) with copy numbers of these three cancer-related genes localized on the same chromosomes (previous studies), both performed on the same samples. As a result, the concordance rate of chromosome 7 number and *EGFR *gene copy number were 65-100%(average: 93%), chromosome 9 and *CDKN2A *gene copy number were 17-100% (average: 81%), and chromosome 11 and *CCND1 *gene copy number were 18-100% (average: 80%). These findings have indicated that chromosome number aberrations may significantly effect on the gene copy number on the same chromosome. However, only a limited number of samples, chromosomes, and genes were investigated in this study. Moreover, there were very large variations in the concordance rate of each sample. For examples, with regard to chromosome 11 and *CCND1 *gene, although the average rate is 80%, the minimum rate is only 18%. Therefore, to clarify the association between CIN and copy number aberrations of these cancer-related genes, further investigations are needed.

Several mechanisms have been proposed to explain the induction of CIN in cancer cells, including defects in chromosomal segregation, telomere stability, cell cycle checkpoint regulation, and repair of DNA damage [14]. Although a large number of genes responsible for CIN have been identified in yeast, only a few such genes have been determined in humans [31-33]. These genes include *hBUB1*, *MAD2*, *BRCA1*, *BRCA2*, and *hCDC4 *[34-38]. Although mutations of these genes have been reported, such defects are rare during carcinogenesis in many kinds of human malignancies. The present findings suggest that the majority of these genetic defects occur as a result of altered expression of known genes that contribute to the development of CIN or mutation in, as yet, unidentified genes. Recently, several studies have indicated that the spindle protein NuMA has been shown to be critical for spindle assembly, and plays a key role in multipolar spindle formation, a frequent cause of CIN in cancer cells. Moreover, the *NUMA1 *gene amplification and overexpression has been reported in oral SCC, suggesting abnormalities of this gene may contribute to the development of CIN in this cancer [39,40]. However, additional examinations are required to clarify the mechanism for CIN in cancer cells.

## Conclusion

The present study has demonstrated that FISH analysis is a sensitive, efficient, and promising method for the evaluation of CIN in oral SCCs. Univariate and multivariate analysis demonstrated that CIN grade was strongly associated with a poor prognosis. Analysis of CIN status using FISH on FNA biopsy specimens may be useful in predicting of recurrence and poor prognosis in patients with oral SCCs.

## Competing interests

The authors declare that they have no competing interests.

## Authors' contributions

HS, NU and TA designed the study. HS, NU, KT and KM carried out the FISH analysis. HS and NU evaluated FISH results and wrote the manuscript. HS performed all the statistical analyses. HS, NU, YO and TA conceived of the study, and participated in its design and coordination and helped to draft the manuscript. All authors read and approved the final manuscript.

## Pre-publication history

The pre-publication history for this paper can be accessed here:

http://www.biomedcentral.com/1471-2407/10/182/prepub
